# Low versus High Fluence Parameters in the Treatment of Facial Laceration Scars with a 1,550 nm Fractional Erbium-Glass Laser

**DOI:** 10.1155/2015/825309

**Published:** 2015-07-07

**Authors:** Hyung-Sup Shim, Dai-Won Jun, Sang-Wha Kim, Sung-No Jung, Ho Kwon

**Affiliations:** Department of Plastic and Reconstructive Surgery, College of Medicine, The Catholic University of Korea, Republic of Korea

## Abstract

*Purpose*. Early postoperative fractional laser treatment has been used to reduce scarring in many institutions, but the most effective energy parameters have not yet been established. This study sought to determine effective parameters in the treatment of facial laceration scars. *Methods*. From September 2012 to September 2013, 57 patients were enrolled according to the study. To compare the low and high fluence parameters of 1,550 nm fractional erbium-glass laser treatment, we virtually divided the scar of each individual patient in half, and each half was treated with a high and low fluence setting, respectively. A total of four treatment sessions were performed at one-month intervals and clinical photographs were taken at every visit. *Results*. Results were assessed using the Vancouver Scar Scale (VSS) and global assessment of the two portions of each individual scar. Final evaluation revealed that the portions treated with high fluence parameter showed greater difference compared to pretreatment VSS scores and global assessment values, indicating favorable cosmetic results. *Conclusion*. We compared the effects of high fluence and low fluence 1,550 nm fractional erbium-glass laser treatment for facial scarring in the early postoperative period and revealed that the high fluence parameter was more effective for scar management.

## 1. Introduction

Facial laceration is one of the most common trauma cases seen in the outpatient department of plastic surgery. Once the laceration is inflicted, a scar formation of some degree is inevitable no matter how meticulously the operator sutures the wound. This may be observed especially in the facial area, due to the active, constantly functioning facial musculature. To minimize the resultant laceration or surgical incision scars, various scar management and prevention methods have been introduced over recent decades, including intralesional steroids, radiotherapy, dermabrasion, pressure therapy, cryosurgery, silicone gel sheeting or ointment massage, and surgical excision [1–5]. However, controversy regarding the most effective method remains, despite there being only a few good treatment options for facial scarring. Recently, due to increased demand for scar management, the efficacy of various methods, including laser treatment, is being evaluated [1–5]. Although various types of lasers are being used, standardized parameters for scar management have yet to be established. Thus, we sought to compare the effects of low and high fluence parameters of 1,550 nm fractional erbium-glass laser treatment for facial scars in the early postoperative period.

## 2. Methods

**Figure 1:**
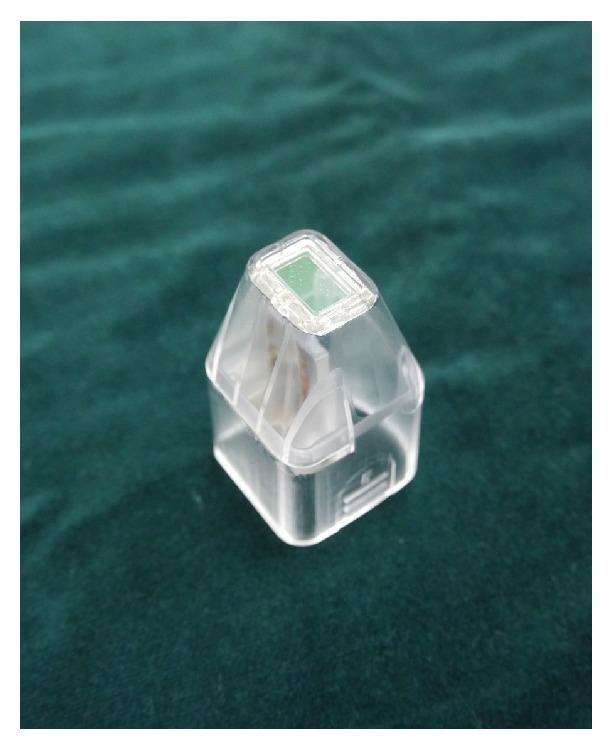
5 × 10 mm handpiece tip was applied along the laceration scar.

This prospective study was designed to establish the most effective fluence parameter. After approval of the Institutional Review Board of Uijeongbu St. Mary's Hospital, a total of 57 patients with Fitzpatrick skin types III–V were enrolled [6]. All patients with facial lacerations primarily sutured in the emergency room by the same senior resident of the plastic surgery department were considered candidates. Patients ranging from 15 to 50 years of age were included. Facial laceration was defined as a laceration within a limited area bordered by the forehead hair line, the preauricular area, and the mandibular angle to the chin. Patients with a facial laceration subcutaneous in depth evenly along the length within 6 cm representing a relatively clean cut without maceration were enrolled. However, patients with acute inflammation, a history of previous trauma, and keloid tendency or those using topical agents including steroids were excluded. Laser treatment began approximately 4 weeks after primary closure. The surface was cleansed with chlorhexidine solution and EMLA cream (a lidocaine-based topical anesthetic cream, AstraZeneca AB, Södertälje, Sweden) was applied for 30 minutes prior to treatment. After removal of the anesthetic cream, the scar was treated with a 1,550 nm fractional erbium-glass laser (MOSAIC HP, Lutronic Co., Ltd., Seoul, South Korea); all patients underwent four sessions at 4-week intervals. In each session, the laceration was virtually divided in half and each half was treated with different parameters: low energy portion (L portion), fluence of 10 mJ/spot, and density of 200 spots/cm^2^; high energy portion (H portion), 50 mJ/spot, and 40 spots/cm^2^. An equivalent amount of energy was delivered to each portion and the parameters and total energy for each session have been set constantly. With 5 × 10 mm handpiece tip (Figure 1), a total of three shots were delivered to each spot without overlap between the spots. The MOSAIC HP automatically starts with skin contact and stops at the end of each session after delivering a set of energy; hence the operator does not need to control the repetition rate or pulse duration.

The results were evaluated at least 6 months postoperatively utilizing two methods. Every photograph was sequentially obtained by a single photographer under identical camera settings (Nikon D600, 24.3 megapixels, Tokyo, Japan) and lighting conditions. First, assessment was performed using the Vancouver Scar Scale (VSS), which included 4 categories: pigmentation (0 = normal, 1 = hypopigmented, 2 = mixed pigmentation, and 3 = hyperpigmented), pliability (0 = normal, 1 = supple, 2 = yielding, 3 = firm, 4 = ropes, and 5 = contracture), height (0 = flat, 1 =≤ 2 mm, 2 = 2–5 mm, and 3 =≥ 5 mm), and vascularity (0 = normal, 1 = pink, 2 = red, and 3 = purple). This was performed by three blinded third-party physicians; each surveyor assigned points for each criterion and from this the total score was calculated. Each portion of the scar in every patient was evaluated separately and VSS score both prior to treatment and after 4 sessions of treatment (6 months postoperatively) was recorded. Second, global assessment was performed by two independent investigators, who evaluated each portion of the scar before and after laser treatment and categorized the outcome as poor, fair, good, or excellent (poor = 1, fair = 2, good = 3, and excellent = 4). Also being difficult to compare the definite global assessment values, the differences of both portions in each patient were checked for comparison.

## 3. Results

**Figure 2:**
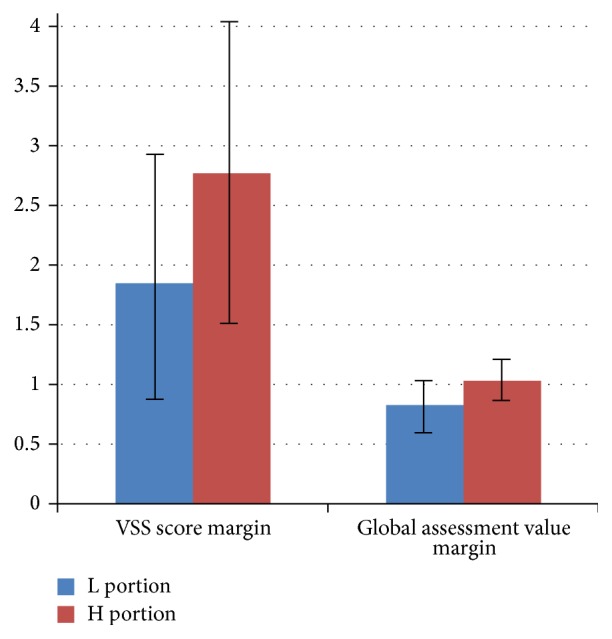
VSS score and global assessment value margins of the two scar portions in each patient. Data are expressed in mean ± standard deviation (*p* < 0.05).

**Figure 3:**
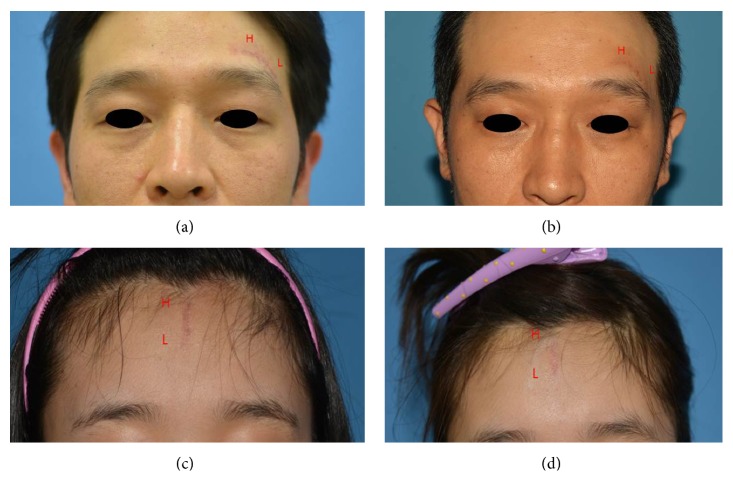
Pre- and posttreatment photography of patients 1 and 2. In patient 1, the horizontal scar of left eyebrow region was divided virtually into the left H portion and right L portion (a). Posttreatment photography (b) one month after the final session showed favorable results in the H portion. In patient 2, the vertical scar of forehead was divided virtually into the upper H portion and lower L portion (c). Posttreatment photography (d) one month after the final session showed favorable results in the H portion.

A total of 57 patients were enrolled. Patient demographics are shown in Table 1. Every patient was evaluated two months after the last treatment session (i.e., 6 months after primary closure). Statistical analyses were conducted using SAS software version 9.3 (SAS institute, Cary, NC, USA) with an independent sample *t*-test, and *p* < 0.05 was considered significant. Because the initial status varied by individual, the difference between pre- and posttreatment scores was used rather than the absolute value for posttreatment score (Figure 2). In every patient, the change observed in the H portion (2.77 ± 1.31) was significantly greater than that seen in the L portion (1.85 ± 1.12) (*p* = 0.038), which suggests that more effective scar management was achieved in the H portion. Global assessment value revealed that the difference of the H portion (1.03 ± 0.18) was significantly greater than the difference of the L portion (0.83 ± 0.21) in every patient (Figure 3). Complications included erythema, inflammation, discharge, and thermal or radiation burn. However, all complications resolved with conservative treatment.

## 4. Discussion

In the past decade, many physicians have reported promising results in treatment of scar using carbon dioxide, pulsed dye, and Er:YAG lasers. Although many have reported that lasers are an effective treatment method for scar management, there have been few studies on nonablative fractional lasers. The mechanism for nonablative fractional laser devices involves the creation of microthermal zones of damage in the treated tissue, extrusion of contents, and rapid reepithelialization within 24 hours allowing for rapid epidermal repair via a rapid migratory path for keratinocytes [7, 8].

One of the studies done by Park et al. sought to determine appropriate initial start timing of nonablative fractional laser treatment of scar [9]. They concluded that early treatment (i.e., within 3 weeks) was more effective than delayed treatment (i.e., within 3 months or within 6 months). As studies of other institutions [9–12], we previously performed a study examining the appropriate period for starting laser treatment of facial laceration scar and concluded similarly that early treatment is more effective. Accordingly, we have come to an idea that varying parameters should affect the final cosmetic result of the early treatment. Thus, this paper investigated appropriate energy parameter settings.

The most troublesome aspect of setting up a laser treatment protocol is how to set the case and control criteria to assess the results. Unlike the study led by Park et al., where only linear scars of surgical incisions made by the same surgeon were targeted, we investigated random laceration scars. After exclusion of dirty ragged scars from the study, we included relatively clear scars and virtually divided them in half to achieve objective comparison between the two parameter settings. In addition, the length of the scar was included in the exclusion criteria because parts of longer scars might be affected differently by facial muscle function, confounding analysis of scar improvement. Before this study, pilot study comparing nontreated patients and fractional laser-treated patients has been performed in our institute. However, we did not elaborate on the result, since the superiority of final cosmetic result in laser-treated patients over nontreated patients has already been proven in many articles [1, 3–5, 9, 11]. Also, the main purpose of our study is to compare treatment parameters; thus we focused on describing the final outcomes of the different treatment parameters.

Setting up a protocol is another challenge. In the case of a 1,550 nm fractional erbium-glass laser, the total energy is sum of the fluence (pulse energy per effective focal area) multiplied by the total density (treatment spots per square centimeter) in every shot. The purpose of this study was not to evaluate total energy delivered but to compare scar improvement depending on different parameters. Thus, we compared low versus high fluence parameters under constant total energy.

Although narrow-band reflectance spectrophotometry is an alternative manner of assessing scars via the erythema index and melanin index, we excluded this method because it assesses only one aspect of the scar. Moreover, the device is too expensive for widespread use in outpatient departments for clinical follow-up.

**Figure 4:**
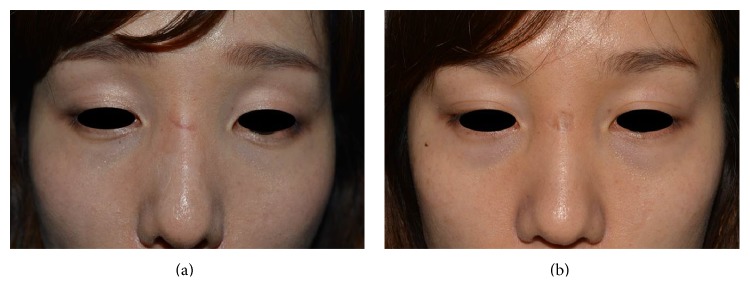
Depressed scar of nose after four treatment sessions with a fluence of 50 mJ/spot ((a) before laser treatment, (b) one month after the final session).

It is possible that parameters greater than 40 mJ/spot might be more effective for reducing scars. However, in our institution, several patients who were not enrolled in this study received deep second degree burns from parameters greater than 40 mJ/spot (Figure 4). In such cases, conservative dressing will result in healing, but depressed scar formation is possible. Accordingly, further study regarding the effects of parameters greater than 40 mJ/spot on impending hypertrophic scars is needed. In addition, different session numbers might also affect scar treatment. Because our study encompasses only facial lacerations meeting certain criteria, scars in different locations or from different injury mechanisms might require distinct protocols.

## 5. Conclusion

Laser therapy is a promising method of scar treatment. Our institution compared the effects of high fluence and low fluence 1,550 nm fractional erbium-glass laser treatment for facial scarring in the early postoperative period and revealed that the high fluence parameter was more effective for scar management. Future studies should investigate the optimal number of sessions or protocols for scars in different locations.

## Figures and Tables

**Table 1:** Demographic data of patients included in the study (M: male, F: female).

Patients	57 (M: 25, F: 32)
Mean age (years)	27.3 (range 15–50)
First laser session (days postoperative)	29.1 (range 27–34)
Follow-up (months after last laser session)	14.7 (range 12–23)
